# Effect of newborn genomic screening for lysosomal storage disorders: a cohort study in China

**DOI:** 10.1186/s13073-025-01483-z

**Published:** 2025-05-12

**Authors:** Xin Wang, Yun Sun, Xian-Wei Guan, Yan-Yun Wang, Dong-Yang Hong, Zhi-Lei Zhang, Ya-Hong Li, Pei-Ying Yang, Tao jiang, Zheng-feng Xu

**Affiliations:** Genetic Medicine Center, Women’s Hospital of Nanjing Medical University, Nanjing Women and Children’s Healthcare Hospital, Qinhuai District, 123 Tianfei Lane, Mochou Road, Nanjing, Jiangsu Province 210004 China

**Keywords:** Lysosomal storage disorder, Newborn screening, Newborn genomic screening, Rare diseases

## Abstract

**Background:**

Lysosomal storage disorders (LSDs) have a relatively high incidence among rare diseases and can lead to severe consequences if not treated promptly. However, many countries and regions have not included these disorders in their newborn screening programs, resulting in missed early detection, underdiagnosis, and delayed treatment. Newborn genomic screening (NBGS) has shown good screening effectiveness for traditional biochemical screening diseases; however, its effectiveness for LSDs has not yet been evaluated in the general newborn population.

**Methods:**

To evaluate the outcome of NBGS for LSDs, a cohort study was conducted involving newborns recruited from Nanjing Women and Children’s Healthcare Hospital in China from March 18, 2022, to September 21, 2023. All participants underwent NBGS of 15 LSDs (18 genes) via dried blood spots, followed by enzyme activity testing for NBGS-positive individuals. The study calculated the incidence and carrier rates for each LSD though NBGS, as well as the positive screening rate, the false positive rate and the positive predictive value of the screening process.

**Results:**

Among 22,687 newborns (11,996 males [52.88%]), 1344 (6.0%) were identified as carriers, and 30 (0.13%) were initially positive for LSDs. Of these, 4 were excluded, 15 were diagnosed as LSD-presymptomatic individuals based on enzyme deficiency and pathogenic variants conforming to inheritance patterns, and 11 remain under follow-up. The estimated combined birth incidence of LSDs in Nanjing was 1/1512, primarily including Fabry disease, Krabbe disease, glycogen storage disease type II, Niemann–Pick disease, and mucopolysaccharidosis type II.

Rather than directly comparing NBGS and enzyme activity screening, this study evaluated two sequential screening strategies: (1) NBGS-first with reflex enzyme testing and (2) enzyme activity-first with reflex genomic testing. The NBGS-first strategy demonstrated higher sensitivity and specificity, with a significantly lower false positive rate and higher positive predictive values compared to the enzyme-first strategy (*P* < 0.05).

**Conclusions:**

This study highlights the potential of NBGS to enhance early detection of presymptomatic LSD individuals, enabling timely interventions and improving newborn health outcomes. Integrating NBGS into routine newborn screening programs could provide an effective and proactive approach for LSD identification and management.

**Supplementary Information:**

The online version contains supplementary material available at 10.1186/s13073-025-01483-z.

## Background

Newborn screening is a highly effective public health initiative that involves widespread screening for specific genetically or congenitally harmful diseases during the newborn phase. The objective is to achieve early diagnosis and treatment, aiming to prevent or mitigate the negative impact of these diseases and enhance the quality of life for families affected by them [1]. Presently, newborn screening predominantly focuses on genetic metabolic disorders due to limitations in detection methodologies [2–5].

Lysosomal storage disorders (LSDs) are among the rare diseases with a relatively high overall incidence and can result in severe clinical outcomes. Early detection, diagnosis, and intervention are critical for effective management. However, routine newborn screening for LSDs has only been adopted in a few regions globally, including parts of the USA [6], the UK [7], northern Italy [8], Japan [9], and Taiwan [10, 11]. In mainland China, voluntary LSD screening has been introduced in specific regions, such as Shanghai [10], Beijing [12], and Shandong [13], but it is not yet widely implemented. Consequently, LSD screening has not been fully incorporated into China’s national newborn screening program, and its adoption varies across regions. Currently, the primary method for screening LSDs involves measuring lysosomal enzyme activity.

Research conducted in various countries and regions indicates that newborn genomic screening (NBGS) has multiple benefits. These include a significant reduction in the false-positive rate associated with traditional newborn screening (tNBS), broader disease coverage, more detailed risk information, and enhanced support for future genetic counseling [14–16]. Moreover, prospective parents generally strongly support the implementation of the NBGS [17–20]. Collectively, these findings suggest that the NBGS has the potential to represent a significant advancement in the field of newborn screening. However, the potential of NBGS for newborn LSDs remains inadequately explored. Consequently, we plan to analyze samples from more than 22,000 newborns to assess the clinical application value of integrating LSDs genomic screening with newborn screening. This study aims to establish a foundation for the clinical screening, diagnosis, and genetic counseling of LSDs.

## Methods

### Study population and data collection

This investigation was conducted at Nanjing Women and Children’s Healthcare Hospital in Jiangsu Province, China. From March 18, 2022, to September 21, 2023, a total of 30 440 infants were born at Nanjing Women and Children’s Healthcare Hospital. From this cohort, 22,687 newborns (11,996 males [52.88%]) participated in the Newborn Genomic Screening (NBGS) program. The cost of the screening was approximately 500 CNY (50 GBP) per case. The demographic characteristics of the study population were as follows: the median gestational age was 39.3 weeks (Q1–Q3: 38.7, 39.3 weeks), and the median birth weight was 3365 g (Q1–Q3: 3082.5–3670 g). Additionally, 9456 newborns (41.69%) were delivered by cesarean section (Table 1). A subset of 900 samples was tested for lysosomal enzyme activity, which included 290 traditional newborn screening negative samples, 29 NBGS-positive samples for LSDs (excluding one MPS II case without enzyme activity testing), 38 NBGS-positive samples for other diseases (Additional file 1: Table S1), and 543 LSD carriers identified through NBGS. The distribution of the LSD carriers included 220 Krabbe, 153 GSD II, 117 NPD-A/B, 45 MPS I, and 8 individuals with multiple gene carriers.

**Table 1:** Characteristics of the newborns in the study cohort

	NBGS	NBGS + Enzyme activity detection				
	No. (%) (*N* = 22,687)	All (*N* = 900)	Negative ^a^ (*N* = 290)	Carrier ^b^ (*N* = 543)	Positive ^c^ (*N* = 29)	Non-LSD Positive ^d^ (*N* = 38)
Sex						
Male	11,996 (52.88)	490 (54.44)	158 (54.48)	290 (53.41)	14 (48.28)	28 (73.68)
Female	10,691 (47.12)	410 (45.56)	132 (45.52)	253 (46.59)	15 (51.72)	10 (26.32)
Unknown	0 (0.00)	0 (0.00)	0 (0.00)	0 (0.00)	0 (0.00)	0 (0.00)
Gestation age						
Preterm (< 37 wk)	857 (3.78)	25 (2.78)	2 (0.69)	19 (3.50)	3 (10.34)	1 (2.63)
Full term (37–40 wk)	16,511 (72.78)	661 (73.44)	220 (75.86)	396 (72.93)	19 (65.52)	26 (68.42)
Late term (40.1–42 wk)	5308 (23.40)	214 (23.78)	68 (23.45)	128 (23.57)	7 (24.14)	11 (28.95)
Post term (> 42 wk)	0 (0.00)	0 (0.00)	0 (0.00)	0 (0.00)	0 (0.00)	0 (0.00)
Unknown	11 (0.05)	0 (0.00)	0 (0.00)	0 (0.00)	0 (0.00)	0 (0.00)
Birth weight						
< 2750 g	1469 (6.48)	44 (4.89)	6 (2.07)	34 (6.26)	3 (10.34)	1 (2.63)
2750–4000 g	21,018 (92.64)	816 (90.67)	276 (95.17)	483 (88.95)	24 (82.76)	33 (86.84)
> 4000 g	190 (0.84)	40 (4.44)	8 (2.76)	26 (4.79)	2 (6.90)	4 (10.53)
Unknown	10 (0.04)	0 (0.00)	0 (0.00)	0 (0.00)	0 (0.00)	0 (0.00)
Delivery						
Eutocia	13,224 (58.29)	500 (55.56)	142 (48.97)	325 (59.85)	11 (37.93)	22 (57.89)
Cesarean	9456 (41.71)	400 (44.44)	148 (51.03)	218 (40.15)	18 (62.07)	16 (42.11)
Unknown	7 (0.03)	0 (0.00)	0 (0.00)	0 (0.00)	0 (0.00)	0 (0.00)

^a^ “Negative” refers to samples with negative NBGS results; ^b^ “Carrier” refers to samples identified as carriers of LSDs based on NBGS results; ^c^ “Positive” refers to samples with an NBGS result indicating LSD positivity; ^d^ “Non-LSD Positive” refers to samples with positive results for non-LSD diseases and have been clinically diagnosed

Ethical approval for the study was granted by the Ethics Committee of the Women’s Hospital of Nanjing Medical University (2021KY-071), and written informed consent was obtained from the parents of the participating newborns.

### Heel blood collection

When the newborn was 48–72 h old and fully fed (either breast milk or formula), a 200 µL heel blood sample was obtained to produce a dried blood filter paper for the detection of disease-associated genes through targeted capture-based next-generation sequencing (NGS).

### NBGS based on targeted capture-based NGS

A total of 94 diseases and 164 genes, including those associated with lysosomal diseases, can be detected using targeted capture-based NGS. A detailed list of the 15 lysosomal diseases and their corresponding 18 genes is provided in Additional file 1: Table S2, with the remaining diseases and genes listed in Additional file 1: Table S3 of the Additional files. Sequence alignment is based on the human genome reference hg19. Genomic DNA was extracted via the QIAamp DNA Blood Midi Kit (51,185, Qiagen, Hilden, Germany), followed by fragmentation via a Covaris LE220 ultrasonic instrument. Magnetic beads were used to isolate fragments (150–200 bp), which were subjected to purification, end repair, A-tailing, and adapter ligation for DNA library construction. Library quantification was performed with an Agilent Bioanalyzer 2100. After A-tailing and ligation, a custom panel of IDT xGen Lockdown probes captured the target sequences, and the hybridization library was sequenced via a high-throughput gene sequencer (MGISEQ-2000) following dynabead creation with a PE100 + 10 sequencing type.

LSDs can be included in newborn screening according to the principle of early disease detection [21–23], which meets the following criteria: (1) the disease can be seriously harmful, and its early symptoms might not be obvious; (2) we have a good understanding of the disease, it occurs somewhat frequently, and without timely intervention, it can lead to poor outcomes; and (3) there are effective treatments available for the disease.

### Bioinformatics analysis

The BasecallLite tool was used to convert raw high-throughput sequencing data from the CAL format to the Fastq format according to protocol of genetic sequencer (MGI). After low-quality reads were filtered out, the sequencing reads were aligned to the NCBI human reference genome (hg19/GRCh37). GATK software was used for the detection of single nucleotide variants and insertions/deletions (indels) [24]. Variant site frequencies in the normal population were obtained from dbSNP (http://www.ncbi.nlm.nih.gov/snp) [25], the 1000 Genomes Project (http://browser.1000genomes.org) [26], and the Exome Aggregation Consortium (http://exac.broadinstitute.org/) [27].

Priority was given to variants meeting either of the following criteria: (1) identified as pathogenic (P) or likely pathogenic (LP) in ClinVar; (2) categorized as nonsense, frameshift, or canonical variants affecting splice sites or the initiation codon in genes known for loss-of-function mechanisms, with an allele frequency of 1% or less in population databases (GnomAD [28], ESP6500 [29], and 1000 Genomes [26]). The interpretation of variants followed the guidelines of the American College of Medical Genetics [30] and literature searches [31–33], considering the level of evidence supporting pathogenicity.

Disease-associated genes and sites were identified and correlated with diseases via databases such as OMIM (http://www.omim.org) [34], ClinVar (http://www.ncbi.nlm.nih.gov/clinvar) [35], and the Human Gene Variant Database (http://www.hgmd.org) [36]. Predictions of the biological functions affected by the variants were made via software, including SIFT (http://sift.jcvi.org) [37], Variant Taster (http://www.varianttaster.org) [38], PolyPhen-2 (http://genetics.bwh.harvard.edu/pph2) [39], and PROVEAN (http://provean.jcvi.org/index.php) [40].

### Sanger sequencing

Positive variants identified by NGS were validated by Sanger sequencing. Genomic DNA extracted from dried blood spots was used to amplify the target region with specific primers. The amplification reaction employed Phanta Max Master Mix (Vazyme, China). The purified PCR products were subjected to sequencing and analysis through capillary electrophoresis, which was conducted with an ABI Prism 3500XL Genetic Analyzer (Thermo Fisher, Waltham, MA, USA).

### Lysosomal enzyme activity detection

The NeoLSD™ MSMS kit (PerkinElmer, Massachusetts, USA) was used for the quantitative measurement of the activity of the lysosomal enzymes acid-β-glucocerebrosidase (ABG), acid-sphingomyelinase (ASM), acid-α-glucosidase (GAA), β-galactocerebrosidase (GALC), α-galactosidase A (GLA), and α-L-iduronidase (IDUA). A 3.2 mm dried blood spot was punched into a 96-well plate and treated with a 30 μL assay cocktail (3093–0020, PerkinElmer, USA). After sealing, the plate was incubated for 18 h at 37 °C with orbital shaking. Following incubation, 100 μL of quenching solvent (methanol/ethyl acetate, 1:1, v/v) was added, and the mixture was transferred to a deep well plate. Liquid‒liquid extraction was performed by adding ethyl acetate and water to each well. After centrifugation, 50 μL of the ethyl acetate phase was transferred to a new plate, and the solvent was evaporated with nitrogen gas. The sample was reconstituted with 100 μL of mobile phase solution (3093–0020, PerkinElmer, USA) before being subjected to LC‒MS/MS analysis via a Waters 1525μ HPLC PUMP and Xevo TQD instrument equipped with a Waters 2777 sample manager.

### NBGS results, diagnosis and follow-up

In this study, a genetically positive result is defined by P/LP variants matching the disease inheritance pattern. A carrier is an individual with a single P/LP variant in autosomal recessive LSD genes, and the carrier rate refers to the proportion of individuals with heterozygous P/LP alleles for each LSD in the screened population, calculated as the number of carriers per total screened newborns (1/n). A negative result refers to individuals with no P/LP variants detected for the LSDs studied.

Newborns with positive NBGS results were recalled for confirmatory enzyme activity testing. In this study, an LSD diagnosis based on newborn screening exclusively refers to a presymptomatic diagnosis, defined as the absence of clinical signs or symptoms while meeting both of the following criteria [41]: (1) Enzyme activity deficiency corresponding to the suspected LSD type; (2) Genetically positive results in disease-associated genes conforming to inheritance patterns. Incidence in this study refers to the identification of new presymptomatic LSD cases through NBGS and enzyme activity testing.

Diagnosed LSDs presymptomatic individuals undergo long-term follow-up, including physical examinations and growth assessments. The specific follow-up intervals and evaluation components vary depending on the type of LSD and are detailed in Additional file 1: Table S4. Personalized follow-up plans are tailored based on clinical findings, parental preferences, and family dynamics to ensure timely intervention when necessary.

### Statistical analysis

Gestational age and birth weight data are presented as median (interquartile range). Statistical analyses were performed using the Kruskal–Wallis test (for non-normally distributed groups), the Mann–Whitney *U* test (for comparisons between two non-normally distributed groups), and paired *t*-tests (for comparisons of positive predictive values, PPV), all using GraphPad version 6.02. *P* values < 0.05 were considered significant (*P < 0.05; **P < 0.01; ***P < 0.001). The Kappa index was calculated with R (version 4.2.2), assessing observed versus expected agreement. Kappa values (κ) indicate the following: 0.01–0.20 (none to slight), 0.21–0.40 (fair), 0.41–0.60 (moderate), 0.61–0.80 (substantial), and 0.81–1.00 (almost perfect agreement).

The evaluation metrics for sensitivity, specificity, false positive rate (FPR), false negative rate (FNR), and PPV are calculated using the following formulas:

- Sensitivity = True Positives / (True Positives + False Negatives)
- Specificity = True Negatives / (True Negatives + False Positives)
- FPR = False Positives / (False Positives + True Negatives)
- PPV = True Positives / (True Positives + False Positives)

The definitions of true positive, true negative, false positive, and false negative in this study are as follows:

- True Positive (NBGS): Cases with positive NBGS results for LSDs (e.g., Krabbe) that were subsequently confirmed as presymptomatic individuals (asymptomatic during newborn screening but meeting genetic and enzyme activity criteria) with the same LSDs.
- True Positive (enzyme activity screening): Cases with positive enzyme activity screening results for LSDs (e.g., Krabbe) that were subsequently confirmed as presymptomatic individuals (asymptomatic during newborn screening but meeting genetic and enzyme activity criteria) with the same LSDs.
- True Negative (NBGS): Cases with negative NBGS results or those identified as carriers of LSDs (e.g., Krabbe) who were assessed to have a low risk of developing the same LSDs.
- True Negative (enzyme activity screening): Cases with negative enzyme activity screening results of LSDs (e.g., Krabbe) that were assessed to have a low risk of developing the same LSDs.
- False Positive (NBGS): Cases with positive NBGS results for LSDs (e.g., Krabbe) that were subsequently determined to have a low risk of developing the same LSDs.
- False Positive (enzyme activity screening): Cases with positive enzyme activity screening results for LSDs (e.g., Krabbe) that were subsequently determined to have a low risk of developing the same LSDs.
- False Negative (NBGS): Cases with negative NBGS results or identified as carriers of LSDs (e.g., Krabbe) but subsequently confirmed as LSD-presymptomatic individuals (asymptomatic during newborn screening but meeting genetic and enzyme activity criteria).
- False Negative (enzyme activity screening): Cases with negative enzyme activity screening results of LSDs (e.g., Krabbe) but subsequently confirmed as LSD-presymptomatic individuals (asymptomatic during newborn screening but meeting genetic and enzyme activity criteria).

## Results

### Screening status for LSDs genes

**Fig. 1:**
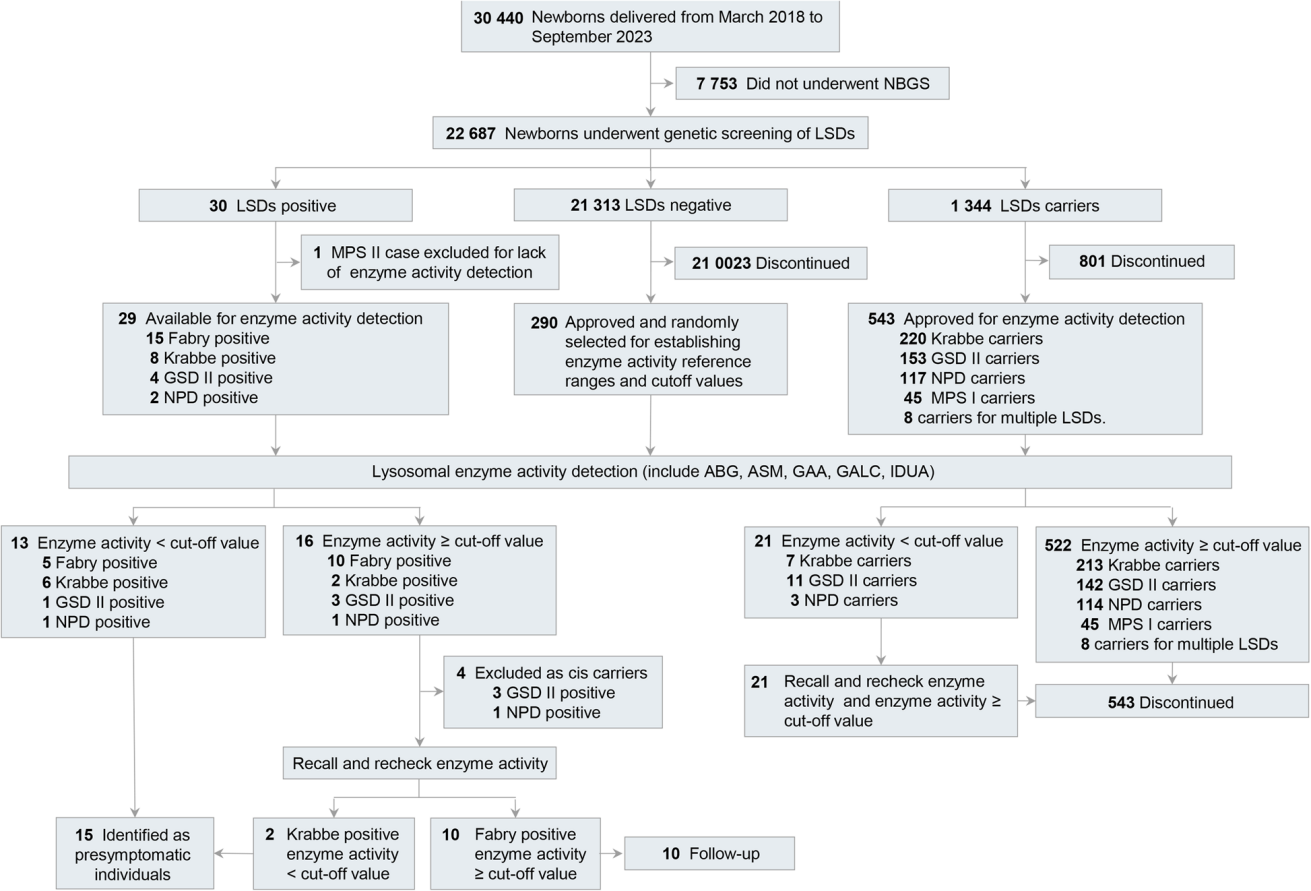
Study flowchart

Among the 22,687 individuals screened, 21,296 tested negatives for LSD gene screening, 1,361 were carriers (24 carriers with two different disease variants), and 30 had initial positive results (Fig. 1). Among the initial positive cases, Fabry disease had the highest proportion (50%) and the highest initial positive rate (0.066%, 1/1512), followed by Krabbe disease (26.67% and 0.035%, 1/2836), glycogen storage disease type II (GSD II) (13.33% and 0.018%, 1/5\672), Niemann‒Pick disease (NPD) (6.66% and 0.009%, 1/11,344), and mucopolysaccharidosis type II (MPS II) (3.33% and 0.004%, 1/22,687). The overall initial positive rate for LSDs was approximately 0.13% (1/756) (Table 2).

**Table 2:** Results from the NBGS of the LSDs

Disease	Negative screening result	Positive screening result	Positive screening rate (1/n)	Presymptomatic individuals identified	Incidence (1/n)	Excluded (Carrier)	Follow-up	FPR	PPV
Fabry	22,672	15	1/1 512	5	1/4 537	0	10	NA	NA
Krabbe	22,679	8	1/2 836	8	1/2 836	0	0	0%	100%
GSD II	22,683	4	1/5 672	1	1/22 687	3	0	0.0132%	25%
NPD	22,685	2	1/11 344	1	1/22 687	1	0	0.0044%	50%
MPS II	22,686	1	1/22 687	0	NA	0	1	NA	NA
All	22,657	30	1/756	15	1/1 512	4	11	0.0177%	50%

NBGS identified a total of 1367 pathogenic variants for LSDs carried by 1344 newborns. The overall carrier rate was approximately 6.03% (1/17), with Krabbe disease having the highest carrier rate (2.56%, 1/39), followed by NPD (1.05%, 1/95) and GSD II (0.86%, 1/116). Among Krabbe disease carriers, the c.1901 T > C variant is highly prevalent, comprising 86.38% of the P/LP variants associated with Krabbe disease. The c.955C > G variant is more prevalent at 65.19% in carriers of the NPD-A/B type, and the most prevalent variant observed in the GSD II type is c.2132_2133delinsGG, accounting for approximately 23.08%. (Additional file 1: Table S5).

### Enzyme activity detection for LSDs

The lysosomal enzyme assay kit is limited to detecting GALC, GAA, ASM, IDUA, and GLA. Lysosomal enzyme activity was detected in a total of 900 samples. Among these, 290 healthy newborns with negative results in traditional newborn screening were utilized to establish high-risk cutoff values (0.2 or 0.3 multiples of the median) [8, 42] and 0.5–99.5 percentile reference ranges for LSDs enzyme activity (Additional file 1: Table S6). The histogram and statistical data illustrating the distribution of enzymatic activity in healthy newborns are displayed in Additional file 1: Fig. S1. No P/LP variants in the LSDs genes were detected in these samples. A cutoff value lower than the high-risk cutoff value, combined with the presence of relevant gene variants, confirmed the diagnosis as a presymptomatic LSD individual.

**Fig. 2:**
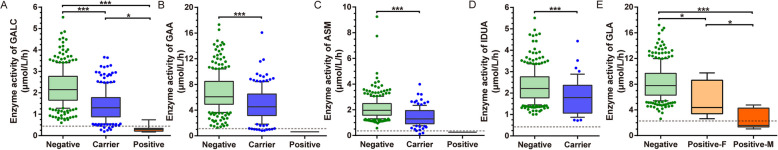
Enzyme activity results for LSD NBGS negative, carriers and positive samples. Enzyme activity of **A** GALC (cutoff value = 0.43 µM/h), **B** GAA (cutoff value = 1.22 µM/h), **C**ASM (cutoff value = 0.39 µM/h), **D** IDUA (cutoff value = 0.44 µM/h), and **E** GLA (cutoff value = 2.33 µM/h). *P < 0.05. ***P < 0.001. The dotted line near the bottom of each figure refers to the cutoff value

The characteristics of the newborns who underwent both NBGS and enzyme activity testing are presented in Table 1, and the detailed information for the 29 NBGS-positive results for LSDs is shown in Table 3. The enzyme activity results for individuals with negative, carrier, and positive NBGS results are illustrated in Fig. 2, which shows a distinct decrease in enzyme activity in positive samples compared with that in negative and carrier samples. Additionally, the carriers presented significantly lower enzyme activity than did the negative samples. There were no statistically significant differences in gestational age or birth weight (Additional file 1: Fig. S2).

**Table 3:** NBGS-positive samples for LSDs

No	Disease	Gene	Genotype	Location	Sex	ACMG variant classification	Enzyme activity (μmol/L/h)	Status	Current age
1	Fabry disease	*GLA*	c.640-801G > A/-	IVS4/-	Male	P	1.91	Presymptomatic LSD	2Y5M
2			c.640-801G > A/-	IVS4/-	Male	P	1.34	Presymptomatic LSD	2Y5M
3			c.640-801G > A/-	IVS4/-	Male	P	1.41	Presymptomatic LSD	2Y8M
4			c.1067G > A/-	EX7E/-	Male	LP	1.57	Presymptomatic LSD	2Y5M
5			c.1067G > A/-	EX7E/-	Male	LP	1.03	Presymptomatic LSD	2Y3M
6			c.911G > C/-	EX6/-	Male	LP	4.76	Follow-up	2Y2M
7			c.911G > C/-	EX6/-	Male	LP	4.27	Follow-up	2Y1M
8			c.640-801G > A/-	IVS4/-	Female	P	3.38	Follow-up	2Y5M
9			c.640-801G > A/-	IVS4/-	Female	P	4.32	Follow-up	1Y10M
10			c.640-801G > A/-	IVS4/-	Female	P	8.62	Follow-up	1Y5M
11			c.640-801G > A/-	IVS4/-	Female	P	3.43	Follow-up	1Y4M
12			c.911G > C/-	EX6/-	Female	LP	9.75	Follow-up	2Y5M
13			c.911G > C/-	EX6/-	Female	LP	8.58	Follow-up	1Y6M
14			c.1072_1074delGAG/-	EX7E/-	Female	LP	4.41	Follow-up	2Y4M
15			c.593 T > C/-	EX4/-	Female	VUS	2.62	Follow-up	1Y11M
16	Krabbe	*GALC*	c.1901 T > C/ c.1912G > A	EX16/EX17E	Male	LP/LP	0.74	Presymptomatic LSD	2Y3M
17			c.1901 T > C/ c.2041G > A	EX16/EX17E	Male	LP/LP	0.21	Presymptomatic LSD	1Y10M
18			c.1901 T > C/ c.1901 T > C	EX16/ EX16	Male	LP/LP	0.29	Presymptomatic LSD	1Y9M
19			c.1901 T > C/ c.1901 T > C	EX16/ EX16	Male	LP/LP	0.34	Presymptomatic LSD	1Y4M
20			c.1901 T > C/ c.1901 T > C	EX16/ EX16	Female	LP/LP	0.17	Presymptomatic LSD	2Y
21			c.1901 T > C/ c.1901 T > C	EX16/ EX16	Female	LP/LP	0.24	Presymptomatic LSD	1Y6M
22			c.1901 T > C/ c.1901 T > C	EX16/ EX16	Female	LP/LP	0.31	Presymptomatic LSD	1Y2M
23			c.1901 T > C/ c.1901 T > C	EX16/ EX16	Female	LP/LP	0.95	Presymptomatic LSD	1Y1M
24	GSD II	*GAA*	[c.2237G > A, c.503G > A]/-	EX16, EX2/-	Male	P/LP	3.51	Excluded	2Y1M
25			[c.2132_2133delinsGG, c.1669A > T]/-	EX11/EX20E	Male	LP/P	3.80	Excluded	1Y11M
26			c.1634C > T/ c.2815_2816delGT	EX15, EX12/-	Male	LP/LP	0.63	Presymptomatic LSD	1Y9M
27			[c.2132_2133delinsGG, c.1669A > T]/-	EX15, EX12/-	Female	LP/LP	0.36	Excluded*	1Y9M
28	NPD-A/B	*SMPD1*	c.995C > G/ c.995C > G	EX2/EX2	Female	LP/LP	0.26	Presymptomatic LSD	2Y
29	NPD-C	*NPC1*	[c.1351G > A, c.3734_3735delCT]/-	EX9/EX24	Female	LP/LP	14.74	Excluded	1Y8M
30	MPS II	*IDS*	c.817C > T/-	EX6/-	Male	LP/-	-	Follow-up	1Y4M

-, no relevant data available. *P* pathogenic, *LP* likely pathogenic. The normal enzyme activity ranges are as follows: for Fabry disease, 3.65–16.60 µM/h; for Krabbe disease, 0.74–4.83 µM/h; for GSD II, 1.45–15.76 µM/h; and for NPD, 0.99–6.45 µM/h. Follow-up indicates long-term monitoring. Excluded refers to individuals with cis-variant arrangement and normal enzyme activity, excluding presymptomatic LSD. Excluded* refers to individuals with a cis-variant arrangement but abnormal enzyme activity, and a recall and recheck of the enzyme activity revealed normal levels (in this case, 1.62 µM/h)

### Incidence and genetic profiles of LSDs

Among the 30 newborns with positive results from the NBGS for lysosomal diseases, 4 were excluded after familial verification and enzyme activity testing. Fifteen newborns were diagnosed as presymptomatic LSD individuals, and 11 newborns were followed-up (10 were Fabry disease NBGS-positive individuals with normal enzyme activity, and 1 was an MPS II NBGS-positive individual, with enzyme activity undetectable using the NeoLSD™ MSMS kit in this study) (Tables 2 and 3). The combined birth incidence rate, which included Fabry disease, Krabbe disease, GSD II, NPD, and MPS II, was 1/1,512, and the diagnosis rate was 0.066%.

There were 15 newborns identified as potential presymptomatic Fabry individuals through NBGS, suggesting a potential overall detection rate of 1/1512. Among them, seven were male, with a male detection rate of approximately 1/1667 (7/11,996), and eight were female, with a female detection rate of approximately 1/1336 (8/10,691). Reduced GLA enzyme activity was found in five males, resulting in a detection rate of 1/4537, lower than the NBGS rate. The primary GLA variant was c.640-801G > A (7/15), followed by c.911G > C (4/15). Eight newborns were presymptomatic individuals of Krabbe disease, with an incidence rate of 1/2782. The primary genotypes included homozygous variations of c.1901 T > C (3/4) and compound heterozygous variations with c.1901 T > C (1/4). There is one presymptomatic individuals each for GSD II, NPD, and MPS II, leading to an incidence rate of 1/22,256 for each of these three diseases.

### NBGS identifies more individuals with presumed presymptomatic LSD than enzyme activity screening

After our statistical investigation, the FPRs and PPVs for the detection of LSD-presymptomatic individuals such as Krabbe disease, GSD II, and NPD through NBGS were obtained, as shown in Table 2. Enzyme activity results in a lack of representativeness in female patients with Fabry disease [43, 44], preventing the evaluation of PPV and FPR.

In the cohort undergoing both NBGS and enzyme activity screening (*n* = 900), we evaluated the sensitivity, specificity, FPR, FNR, and PPV for each screening method, with NBGS and enzyme activity screening serving as the first-tier methods, respectively. It is important to note that in this study, these metrics are specifically used to assess the detection capability for presymptomatic LSD individuals, rather than those with clinically diagnosed LSDs exhibiting significant symptoms. The Kappa results indicated that NBGS outperformed enzyme activity screening in identifying presymptomatic individuals, with a significantly higher PPV for NBGS compared to enzyme activity screening (*P* < 0.05) (Table 4).

**Table 4:** FRP and PPV in NBGS and Enzyme activity screening group

Disease	Enzyme activity-first strategy			NBGS-first strategy		
	Krabbe	GSD II	NPD-A/B	Krabbe	GSD II	NPD-A/B
True negative	885	888	896	892	896	898
False negative	2	0	0	0	0	0
True positive	6	1	1	8	1	1
False positive	7	11	3	0	3	1
False positive (exclude NBGS carrier)	2	2	2	-	-	-
Sensitivity	75%	100%	100%	100%	100%	100%
Specificity	99.22%	98.78%	99.67%	100%	99.67%	99.89%
Specificity (exclude NBGS carrier)	99.77%	99.78%	99.78%	-	-	-
FPR	0.78%	1.22%	0.33%	0%	0.33%	0.11%
FPR (exclude NBGS carrier)	0.23%	0.22%	0.22%	-	-	-
FNR	25%	0%	0%	0%	0%	0%
PPV	46.15%	8.33%	25%	100%	25%	50%
PPV (exclude NBGS carrier)	75%	33.33%	33.33%	-	-	-
Kappa index	0.5667	0.1521	0.3989	1	0.3989	0.6662

The metrics are based on the diagnosis of presymptomatic individuals. -, no relevant data available

These findings suggest that NBGS provides a more proactive screening strategy for identifying LSD-presymptomatic individuals compared to enzyme activity screening when used as a first-tier strategy.

### Understanding factors influencing lysosomal enzyme activity through the NBGS

Among carriers of LSDs, approximately 50% exhibit enzyme activity within the 10–20th percentile of negative samples, whereas 70–85% fall within the 50th percentile (Additional file 1: Table S7). This finding indicates that lysosomal enzyme activity in carriers of disease-associated genes for LSDs often decreases to half of the levels observed in the general population, with approximately half of the carriers displaying even lower enzyme activity, which may increase the likelihood of false-positive results in lysosomal activity testing for carriers.

**Fig. 3:**
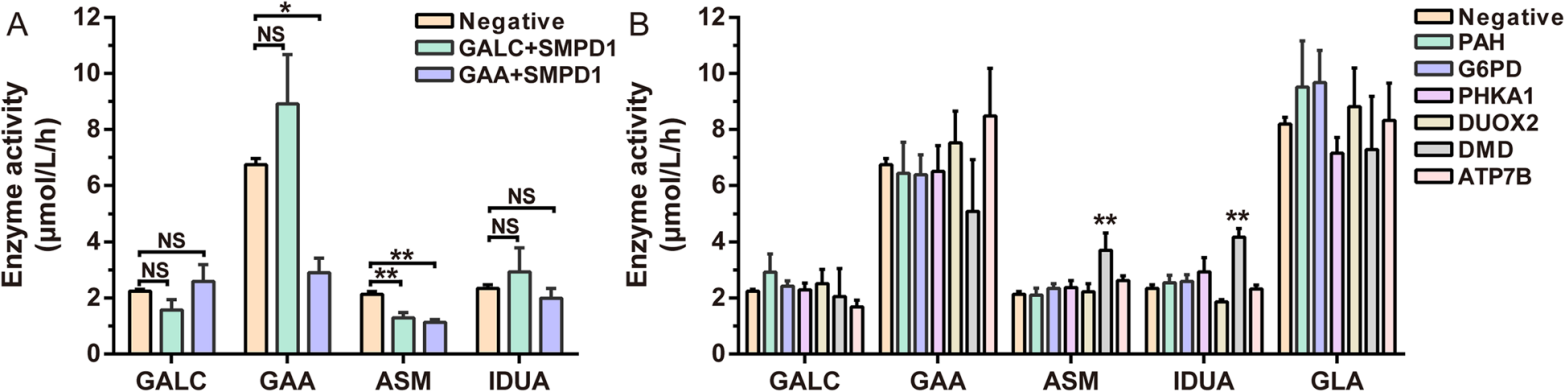
Influencing factors on lysosomal enzyme activity. **A** Lysosomal enzyme activity in carriers of multiple P/LP variants for LSDs. GALC + SMPD1: individuals with P/LP variants in both GALC and SMPD1. GAA + SMPD1: individuals with P/LP variants in both GAA and SMPD1. **B** Lysosomal enzyme activity in newborns with positive NBGS results for other diseases. PAH: hyperphenylalaninemia-related gene. G6PD: glucose-6-phosphate dehydrogenase deficiency gene. PHKA1: glycogen storage disease IX d-related gene. DUOX2: congenital hypothyroidism-related gene. DMD: Duchenne muscular dystrophy-related gene. ATP7B: hepatolenticular degeneration-related gene. NS, not significant. *P < 0.05; **P < 0.01

Moreover, carriers with multiple gene variants that are P/LP for LSDs show a reduction in the corresponding enzyme activities of each gene (Fig. 3A). When carriers have both disease-associated genes for LSDs and genes for other conditions, such as those identified in traditional newborn screening or diseases lacking biochemical testing methods, lysosomal enzyme activity remains unaffected, which indicates that carrier P/LP variants for other diseases may not necessarily impact the detection of lysosomal enzyme activity (Additional file 1: Fig. S3). Testing lysosomal enzyme activity in positive cases from NBGS for several common newborn genetic disorders revealed an increase in ASM and IDUA enzyme activity in infants with Duchenne muscular dystrophy (DMD), indicating a greater likelihood of false positives in ASM and IDUA enzyme activity for DMD infants (Fig. 3B).

## Discussion

To explore the significance and clinical application of NBGS for LSD screening, we conducted prospective NBGS on 22,687 newborns and selected 900 newborns for lysosomal enzyme activity testing to compare the two screening strategies preliminarily. To our knowledge, this study is the first to focus on the role of NBGS in LSD screening and compare it with current enzymatic screening methods, aiming to evaluate the potential benefits of applying NBGS to LSD screening.

Our prospective cohort study revealed that among the 22,687 newborns screened with NBGS, 30 were positive, with an initial screening positivity rate of 0.13%. Four cases were excluded after familial validation, resulting in a false positive rate of 0.0177%, calculated based on the diagnosis of presymptomatic LSD individuals. This rate is considerably lower than the false positive rate reported for enzyme activity screening [45]. Fifteen cases were confirmed as presymptomatic individuals, with a PPV of approximately 50%. The overall incidence is 1/1512, much higher than the incidence estimated on the basis of enzyme activity screening statistics [45–47], indicating that NBGS-based newborn LSD screening may identify more presymptomatic LSD individuals compared to enzyme activity screening. Additionally, approximately one presymptomatic individuals of LSD per 1,512 newborns may benefit from timely disease tracking management and intervention through the NBGS.

The incidence of LSDs reported in our study may be greater than that previously reported in clinically symptomatic populations [47]. This difference arises because earlier statistics primarily included patients who had already developed symptoms and sought medical attention, potentially overlooking individuals who are asymptomatic, misdiagnosed, or have mild symptoms and did not seek medical care. In contrast, the NBGS can detect presymptomatic individuals who have not yet exhibited obvious clinical symptoms. A recent study on newborn screening for LSDs on the basis of lysosomal enzyme activity in the Shanghai region of China reported an incidence similar to that reported in our study [10].

LSDs encompass a wide range of diseases, with up to 70 types [47]. Currently, LSDs clinically screened through enzyme activity mainly include four to six types, such as Fabry disease, GSD II, NPD, Gaucher disease, mucopolysaccharidosis type I, and Krabbe disease. We propose a screening approach that uses genomic testing as first-tier screening supplemented by enzymatic testing. The conditions included are not all LSDs but 15 specific LSDs selected after comprehensive evaluation according to the W&J criteria [21], expanding the scope of screened diseases compared with enzyme activity screening. Each of these 15 LSDs has a clear diagnostic method, primarily combining enzymatic and genetic testing, which allows for presymptomatic diagnosis. Furthermore, all 15 LSDs have treatment options available (Additional file 1: Table S2), including symptomatic and supportive therapy, small molecule therapy, substrate reduction therapy, enzyme replacement therapy, and gene therapy [47, 48].

Newborn screening for LSDs plays a crucial role in early detection, significantly reducing diagnostic delays and preventing irreversible organ damage before clinical symptoms manifest. Given the rarity of LSDs, many cases go unrecognized until significant disease progression has occurred [41]. In this study, presymptomatic individuals identified through NBGS and enzyme activity testing had not yet exhibited overt clinical symptoms. However, early detection of such individuals is critical for LSD management. Long-term follow-up allows for monitoring disease progression and determining optimal intervention timing, particularly for late-onset LSDs such as Pompe and Fabry disease. Proactive health management facilitates timely medical decision-making and helps prevent severe disease manifestations (Additional file 1: Table S4). Additionally, NBGS supports genetic counseling and cascade screening, enabling at-risk family members to receive early assessment and necessary interventions. Regular follow-up of presymptomatic individuals ensures early detection of disease progression, leading to timely clinical interventions and improved prognoses. The established consensus on long-term follow-up for newborns testing positive for Krabbe disease serves as a reference for managing other LSDs [49], highlighting the necessity of early screening and continuous monitoring. Without newborn screening, many patients remain undiagnosed until severe symptoms prompt medical attention, limiting treatment effectiveness. Thus, NBGS not only facilitates systematic health management for presymptomatic individuals but also contributes to healthier and more secure lives.

In newborn screening, key metrics such as FPR, FNR, PPV, sensitivity, and specificity are essential for evaluating the clinical utility of the screening process, as they directly impact both the accuracy of results and subsequent clinical decisions. In this study, FPR refers to the proportion of healthy newborns incorrectly identified as presymptomatic individuals. A high FPR can lead to unnecessary testing, increasing anxiety and straining resources. FNR reflects the proportion of LSD-presymptomatic individuals who are incorrectly classified as healthy. A high FNR can result in missed diagnoses of presymptomatic individuals, delaying timely interventions. PPV indicates the likelihood that a newborn with a positive screening result is truly a presymptomatic individual. A higher PPV enhances the reliability of the screening results. Sensitivity measures the ability of the screening method to correctly identify presymptomatic LSD newborns. Higher sensitivity reduces the chances of missed diagnoses. Specificity evaluates the ability of the screening to accurately identify healthy newborns. High specificity helps to reduce false positives, thereby minimizing unnecessary follow-up testing. By comparing these evaluation metrics, we evaluated two screening strategies within the same population: NBGS as the first-tier screening and enzyme activity screening as the first-tier approach. Our findings show that NBGS was more effective in identifying presymptomatic LSD individuals as a first-tier screening strategy (Table 4). A review of previous domestic and international studies on enzyme activity-based screening for LSDs further suggests that NBGS may have greater potential as a first-tier screening strategy for LSDs such as Krabbe disease, GSD II, and NPD-A/B, particularly in Asian regions like Japan and China [6, 8, 10, 11, 50–57]. However, variations in evaluation metrics due to regional, ethnic, and diagnostic criteria differences should be considered (Additional file 1: Table S8). Additionally, we found that excluding carrier samples from enzyme activity screening improved all metrics compared to when carriers were included (Table 4 and Additional file 1: Table S7). This suggests that carrier samples may interfere with enzyme activity testing. Beyond carrier interference, enzyme activity screening may also be influenced by other factors, such as pseudo-defective alleles (No. 26 in Table 3) or confounding effects from other diseases (Fig. 3). NBGS, based on genetic analysis, effectively addresses the limitations of enzyme activity-based LSD screening, minimizing interference from external factors such as carriers.

The onset of clinical symptoms varies across different LSDs and even among patients with the same LSD due to genotypic differences, with some cases being mild or late-onset and not appearing in the neonatal period (Additional file 1: Table S9). NBGS enables close monitoring of presymptomatic individuals with LSDs exhibiting varying onset times. For instance, the potential cases identified in this study currently display only abnormal enzyme activity, with no other disease-related clinical symptoms. The oldest presymptomatic individuals is currently 2 years and 8 months old. Given their young age, follow-up focuses on physical exams and growth assessments every 6 to 12 months, with no abnormalities observed to date. According to national and international guidelines for the management of these diseases [49, 58–63], we recommend initiating comprehensive follow-up assessments in childhood that encompass evaluations of the renal, cardiac, respiratory, and nervous systems, as well as laboratory tests, with follow-up intervals based on clinical findings and timely initiation of treatment when indicated (Additional file 1: Table S4). Additionally, the actual follow-up plan should be customized to the individual circumstances of each child, taking into account parental preferences and family dynamics. NBGS for LSDs with varying onset times not only allows close monitoring of presymptomatic individuals but also provides insights into family health and aids in genetic counseling. For example, for X-linked diseases such as Fabry disease, NBGS can trace affected family members after identifying potential presymptomatic individuals and enabling timely detection and intervention. For autosomal recessive disorders, NBGS can reveal carrier status in family members, helping to assess genetic risk and optimize genetic counseling [64].

Although our research suggests that NBGS is more effective than enzyme activity screening as a first-tier method for presymptomatic LSD individuals, further optimization of the screening strategy is still needed. Currently, established LSDs screening kits can only detect five of the fifteen high-prevalence disorders. For the remaining ten LSDs, while we have developed our own methods for enzyme activity or metabolite detection, these methods are not based on tandem mass spectrometry and are suitable only for secondary screening or confirmatory diagnosis in presymptomatic individuals, not large-scale screening. Therefore, we can only test individuals who are positive for NBGS, which carries a risk of false negatives. To address this, we propose integrating enzyme activity testing with NBGS for the five high-prevalence LSDs to reduce missed cases and false positives. Additionally, developing biochemical screening methods capable of detecting more LSDs through enzyme activity or specific metabolites should be explored. By integrating these approaches with NBGS, we aim to improve the overall effectiveness of LSD detection in presymptomatic individuals. Given that widespread implementation of lysosomal enzyme activity screening may take some time, it may be more practical to prioritize NBGS as the first-tier test, followed by enzyme activity screening as a second-tier test, which could help reduce FPR and improve the PPV. On the basis of optimizing screening methods, more attention should be given to improving disease management after screening, standardizing the follow-up process for presymptomatic individuals to facilitate timely and effective intervention, and improving the quality of life of affected infants. Additionally, considering that many LSDs may be asymptomatic in the neonatal period, in future work, if we can establish connections between the genotypes, enzyme activities, disease severity, and onset time of these diseases through long-term follow-up, we can use genotype and enzyme activity to assess disease severity and approximate onset time, which will further improve the screening, diagnosis, and treatment processes of LSDs.

Previous studies have discussed the advantages of targeted capture-based NGS technology over techniques such as whole-genome sequencing and whole-exome sequencing, citing its lower cost, relatively easy operation, shorter reporting cycle, and easier interpretation [15, 65], making it the most suitable technology for newborn screening at present. However, with further optimization of the techniques and continuous reduction in costs, whole-genome sequencing may become a better alternative in the future.

In this study, 30,440 newborns were eligible for NBGS, and 22,687 (about 74.5%) participated. However, 25.5% of parents declined to have their newborns screened. The main reasons for refusal were as follows: (1) The NBGS is a paid service, costing approximately 850 CNY (92 GBP). While this price is relatively affordable, some families, due to financial constraints, still find it difficult to cover the cost. (2) Some parents, due to limited education, lack sufficient understanding of genetics and NBGS. As a result, they may not fully appreciate the importance of the screening and are less willing to participate. (3) Certain parents had already undergone carrier screening during pregnancy and believe that it covers the same areas as NBGS, thus seeing no need for further testing. Additionally, previous research has shown that while most parents view NBGS positively, some have concerns. These include worries about the potential strain on family relationships or the possibility of social discrimination following the results [17, 66]. This highlights several areas that need to be strengthened when promoting NBGS nationwide: (1) Further reduce the cost of testing or integrate it into health insurance coverage; (2) Strengthen public education to increase awareness among childbearing families about the importance of NBGS, thereby improving participation rates; (3) Ensure strict data security to prevent any leakage of results, making sure that only parents have access to the information; (4) Enhance genetic counseling and follow-up services, providing families with high-quality support to better understand and manage the potential implications of the results, which can help reduce emotional stress and improve overall quality of life for affected families; (5) Enhance the technical training of clinical personnel to improve operational competencies and establish necessary technical platforms to strengthen the screening capacity of healthcare institutions across various regions; 6. Conducting more large-scale studies with longer follow-up, comprehensive confirmatory testing, and better understanding of how many screen-positive individuals actually develop clinical signs or symptoms of LSD, and at what age, to justify national integration of NBGS for LSDs; 7.Develop a national NBGS information platform to facilitate technical exchange and optimize resource integration.

This study has several key limitations. First, its sample size and geographical scope are restricted, providing only a partial understanding of lysosomal disease epidemiology. Future research should consider broader, multicenter newborn screening to increase data reliability. Second, although the NBGS encompasses all coding regions of lysosomal diseases, only variants classified as P/LP according to ACMG guidelines are reported to simplify clinical interpretation and reduce anxiety for families of newborns. However, this approach may lead to a risk of false negatives. For instance, NBGS may identify a newborn as a carrier, but if there is another unreported pathogenic variant, the newborn may actually be at risk. Additionally, this approach may also lead to false positives, as the true penetrance of the variants is unknown. Furthermore, some lysosomal diseases are not suitable for NBGS because of homologous sequences and other reasons. This suggests that future screening efforts should integrate multiple methods rather than relying on a single approach to enhance screening effectiveness. Finally, newborns identified as positive may not exhibit symptoms at the time of screening, which limits our immediate understanding of their condition. However, this study primarily focuses on the ability of NBGS to detect presymptomatic LSD individuals. Early detection, combined with regular follow-up and increased family awareness, can facilitate timely diagnosis and intervention. To further understand the long-term impact, we remain committed to monitoring these newborns over time, gaining deeper insights into their clinical progression and outcomes.

## Conclusions

This study highlights that NBGS is effective in screening for LSDs and can significantly increase the detection rate of LSDs presymptomatic individuals. Early detection of these presymptomatic individuals is essential for providing healthcare and genetic counseling, and it allows newborns and their families to benefit from timely treatment and monitoring. Our study provides a foundation for the clinical application of NBGS in LSDs screening and lays the groundwork for further research in this field.

## Supplementary Information

Additional file 1: Supplementary Tables and Figs.

## Data Availability

The genotype data from the Chinese population cannot be submitted to publicly available databases because the ethical approval did not permit the sharing of raw genotype data. However, the data may be made available upon reasonable request to the corresponding author, in accordance with Chinese genomic data sharing policies. To ensure responsible use, requesters are required to submit a detailed research proposal along with their institutional affiliation. The proposal will be reviewed by the Ethics Committee of the Women’s Hospital of Nanjing Medical University and the Ministry of Science and Technology of the People’s Republic of China. Upon approval and completion of a data use agreement, the dataset will be shared within 30 days.

## Abbreviations

LSDs: Lysosomal storage disorders
NBGS: Newborn genomic screening
tNBS: Traditional newborn screening
ABG: Acid-β-glucocerebrosidase
ASM: Acid-sphingomyelinase
GAA: Acid-α-glucosidase
GALC: β-Galactocerebrosidase
GLA: α-Galactosidase A
IDUA: α-L-iduronidase
GSD II: Glycogen storage disease type II
NPD: Niemann‒Pick disease
MPS II: Mucopolysaccharidosis type II
FPRs: False positive rates
FNRs: False negative rates
PPVs: Positive predictive values
