# Supplementary material for: Effect of newborn genomic screening for lysosomal storage disorders: a cohort study in China
Source: Genome Med. 2025 May 12;17:52. doi: 10.1186/s13073-025-01483-z (PMC12070756; doi:10.1186/s13073-025-01483-z)
Supplement: Supplementary file 1 — Additional file 1: Supplementary Tables and Figs. [file 13073_2025_1483_MOESM1_ESM.pdf]

Additional file 1

Table S1: 38 positive samples for other diseases.

No.	Disease (Gene, Inheritance pattern)	Genotype	Location	Gender	Pathogenetic	Biochemical indicator [a]
1	Hyperphenylalaninemia (*PAH*, AR)	c.721C>T/ c.1045T>G	EX7/EX10	female	P/LP	PHE=152.46
2		c.532G>A/c.505C>A	EX6/EX4	female	LP/LP	PHE=89.09
3		c.1238G>C/c.722delG	EX12/EX7	male	P/P	PHE=538.43
4		c.482T>C/c.158G>A	EX5/EX2	male	LP/VUS	PHE=158.67
5		c.1238G>C/c.158G>A	EX12/EX2	female	P/VUS	PHE=98
6		c.331C>T/c.482T>C	EX3/EX5	male	P/LP	PHE=581.68
7		c.320A>G/c.721C>T	EX3/EX7	female	LP/P	PHE=185.68
8		c.1174T>A/c.1238G>C	EX11/EX12	female	LP/P	PHE=156.03
9		c.611A>G/c.158G>A	EX6/EX2	female	P/VUS	PHE=127.92
10		c.442-1G>A/c.975C>G	IVS4/EX10	male	P/P	PHE=625
11	Congenital hypothyroidism (*DUOX2*, AR)	c.2048G>T/c.2654G>A	EX17/EX20	male	LP/P	TSH=14.62
12		c.2635G>A/c.2654G>A	EX20/EX20	female	P/P	TSH=4.68 [b]
13		c.2654G>T/c.3329G>A	EX20/EX25	male	LP/LP	TSH=8.76
14	Ornithine transcarbamylase deficiency (*OTC*, XL)	c.385C>T/-	EX4/-	male	LP	CIT=12.91
15		c.148G>A/-	EX2/-	female	LP	CIT=15.47

16	2-methylbutyrylglycinemia (*ACADSB*, AR)	c.655G>A/ c.655G>A	EX5/EX5	female	LP/LP	C5=0.38 [c]
17	Very long chain acyl-CoA dehydrogenase deficiency (*ACADVL*, AR)	c.1406G>A/c.637G>A	EX14/EX8	male	LP/LP	C14:1=2.96
18	Glucose-6-phosphate dehydrogenase deficiency (*G6PD*, XL)	c.1024C>T/-	EX9/-	male	LP/-	EA = 2.51
19		c.1376G>T/-	EX12/-	male	LP/-	EA=0.92
20		c.1024C>T/-	EX9/-	male	LP/-	EA=2.00
21		c.392G>T/-	EX5/-	male	P/-	EA=2.30
22		c.1376G>T/-	EX12/-	male	LP/-	EA=0.49
23		c.1360C>T/-	EX11/-	male	LP/-	EA=0.40
24		c.1388G>A/-	EX12/-	male	LP/-	EA=1.72
25		c.95A>G/-	EX2/-	male	LP/-	EA=0.76
26		c.1388G>A/-	EX12/-	male	LP/-	EA=0.73
27	Glycogen storage disease IX d (*PHKA1*, AR)	c.2051delG/-	EX19/-	male	LP/-	-
28		c.2218C>T/-	EX20/-	male	LP/-	-
29		c.1989_1990delinsAAGTTG	EX19/-	male	P/-	-

		CTCGTGATCTAAA/-				
30		c.748_749insT/-	EX8/-	male	LP/-	-
31		c.1039C>T/-	EX10/-	male	LP/-	-
32		c.1989_1990delinsAAGTTG CTCGTGATCTAAA/-	EX19/-	male	P/-	-
33		c.2091C>A/-	EX19/-	male	LP/-	-
34	Duchenne muscular dystrophy (*DMD,* XLR)	EX49_51 DEL/-	EX49_51/-	male	P/-	-
35		EX1_9 DUP/-	EX1_9/-	male	VUS/-	-
36	Hepatolenticular degeneration (*ATP7B*, AR)	c.2804C>T/ c.2297C>T	EX12/EX8	male	LP/LP	-
37		[c.588C>A, c.3316G>A]/ c.2333G>T	EX2, EX15/EX8	male	LP, LP/P	-
38	Hereditary fructose intolerance (*ALDOB*, AR)	c.10C>T/c.524C>A	EX2/EX5	female	P/LP	-

a, reference ranges of the biochemical indicators, PHE 25~90 μmol/L, TSH≤ 7.9 mIU/L, CIT 6~32 μmol/L, C5 0.04~0.35 μmol/L, C14:1 0.03~0.24 μmol/L, EA (G6PD enzyme activity) ≥2.5U/gHb. b, the follow-up TSH result is11.49 mIU/L. c, the follow-up C5 result is 1.05μmol/L.

Table S2: Genomic screening LSDs and genes.

No.	Disease	Gene	Genetic model	Diagnostic method	Treatment Advances
1	MPS I	*IDUA*	AR	Enzyme activity testing + Genetic analysis	ERT, SCT, ST
2	MPS II	*IDS*	XLR	Enzyme activity testing + Genetic analysis	ERT, SCT, ST

3	MPS III (A/B/C)	*SGSH/ NAGLU/ HGSNAT*	AR	Enzyme activity testing + Genetic analysis	ST, ERT/GT Clinical trials
4	MPS IV A	*GALNS*	AR	Enzyme activity testing + Genetic analysis	ERT, ST
5	MPS VI	*ARSB*	AR	Enzyme activity testing + Genetic analysis	ERT, SCT, ST
6	MPS VII	*GUSB*	AR	Enzyme activity testing + Genetic analysis	ST, ERT Clinical trials
7	GSD II	*GAA*	AR	Enzyme activity testing + Genetic analysis	ERT, ST
8	Metachromatic leukodystrophy	*ARSA*	AR	Enzyme activity testing + Genetic analysis	SCT, ST, GT Clinical trials
9	GM1 gangliosidosis	*GLB1*	AR	Enzyme activity testing + Genetic analysis	ST, GT Clinical trials
10	Sandhoff disease	*HEXB*	AR	Enzyme activity testing + Genetic analysis	ST, ERT/GT Clinical trials
11	Tay-Sachs disease	*HEXA*	AR	Enzyme activity testing + Genetic analysis	ST, ERT/GT Clinical trials
12	Krabbe disease	*GALC*	AR	Enzyme activity testing + Genetic analysis	SCT, ST, GT Clinical trials
13	Fabry disease	*GLA*	XLD	Enzyme activity testing + Genetic analysis	ERT, ST, GT Clinical trials
14	NPD-A/B	*SMPD1*	AR	Enzyme activity testing + Genetic analysis	SCT, ST, ERT Clinical trials
15	NPD-C	*NPC1/ NPC2*	AR	FILIPIN staining + Genetic analysis	SRT, ST

Abbreviations: MPS, mucopolysaccharidosis; GSD II, glycogen storage disease type II; NPD, Niemann-Pick disease; AD, autosomal dominant inheritance; AR, autosomal recessive inheritance; XLD, x-linked dominant inheritance; XLR, x-linked recessive inheritance. ERT, enzyme replacement therapy; SRT, substrate reduction therapy; SCT, stem cell therapy; GT, gene therapy; ST, support therapy.

Tabel S3: Genomic screening diseases and genes list (excluding the LSDs).

Category	Disease	Gene	Genetic model
Amino acid metabolism disorders	Maple syrupurine disease (MSUD)	*BCKDHA*	AR
		BCKDHB	AR
		DBT	AR
	Tyrosinemia	*FAH*	AR
		TAT	AR
		HPD	AR
	Hyperphenylalaninemia (HPA)	*PAH*	AR
		PTS	AR

		QDPR	AR
	Homocysteinemia (HCY)	*CBS*	AR
		MTHFR	AR
	Non-ketotic hyperglycinemia (NKH)	*GLDC*	AR
		AMT	AR
	Hypermethioninemia	*MAT1A*	AR/AD
	Hyperprolinemia type 1	*PRODH*	AR
Urea cycle disorders	Argininemia	*ARG1*	AR
	Argininosuccinic aciduria (ASA)	*ASL*	AR
	Carbamoyl phosphate synthetase I deficiency	*CPS1*	AR
	Ornithine transcarbamylase deficiency (OTCD)	*OTC*	XLR
	Citrullinemia	*ASS1*	AR
		SLC25A13	AR
	Hyperornithinemia-hyperammonemia-homocitrullinuria syndrome (HHHS)	*SLC25A15*	AR
Organic acid metabolism disorders	Methylmalonic acidemia (MMA)	*MMAA*	AR
		MMAB	AR
		MMACHC	AR
		MMUT	AR
	Propionic acidemia (PA)	*PCCA*	AR
		PCCB	AR
	Glutaric Acidemia I (GA-1)	*GCDH*	AR
	Holocarboxylase synthase deficiency (HCS)	*HLCS*	AR
	Biotinase deficiency (BTDD)	*BTD*	AR
	3-hydroxy-3-methylglutaryl-coenzyme A lyase deficiency (HMGCL)	*HMGCL*	AR
	Isovaleric acidemia (IVA)	*IVD*	AR
	3-methylcrotonyl-coenzyme A carboxylase deficiency (MCCD)	*MCCC1*	AR
		MCCC2	AR
	Malonyl-coenzyme A decarboxylase deficiency	*MLYCD*	AR
	3-methylpentolenoacidemia type I	*AUH*	AR
	2-methylbutyrylglycinemia	*ACADSB*	AR
	Isobutyryl-coenzyme A dehydrogenase deficiency	*ACAD8*	AR
	β-ketothiolase deficiency	*ACAT1*	AR
	Succinate hemialdehyde dehydrogenase deficiency (SSADHD)	*ALDH5A1*	AR
Fatty acid β oxidation disorders	Very long chain acyl-CoA dehydrogenase deficiency (VLCADD)	*ACADVL*	AR
	Long-chain 3-hydroxyacyl-CoA dehydrogenase deficiency (LCHAD)	*HADHA*	AR
	Trifunctional protein deficiency (TFPD)	*HADHA*	AR

		HADHB	AR
	Medium chain acyl-CoA dehydrogenase deficiency (MCAD)	*ACADM*	AR
	Short-chain acyl-CoA dehydrogenase deficiency (SCADD)	*ACADS*	AR
	Glutaric acidemia type II (GA-2)	*ETFDH*	AR
	Primary carnitine deficiency (PCD)	*SLC22A5*	AR
	Carnitine palmitoyltransferase deficiency (CPT)	*CPT1A*	AR
		CPT2	AR
	Carnitine-acylcarnitine translocase deficiency (CACT)	*SLC25A20*	AR
	2,4-Dienoyl-CoA reductase deficiency	*NADK2*	
Carbohydrate metabolism disorders	Glycogen storage disease (GSD)	*G6PC*	AR
		SLC37A4	AR
		GAA	AR
		AGL	AR
		GBE1	AR
		PYGM	AR
		PYGL	AR
		PHKA2	AR
		PHKB	AR
		PHKG2	AR
		PHKA1	AR
		PGM1	AR
	Galactosemia	*GALK1*	AR
		GALT	AR
		GALE	AR
Lipid metabolism disorders	Sitosterolemia	*ABCG8*	AR
		ABCG5	AR
	Familial hypercholesterolemia type 1 (FH)	*LDLR*	AD
Hematological system diseases	α-thalassemia	*HBA1/HBA2*	AR
		HBA1	AR
		HBA2	AR
	β-thalassemia	*HBB*	AR
	Diamond-Blackfan anemia	*RPS19*	AD
		RPL11	AD
		RPS26	AD
	Fanconi anemia complementary group A	*FANCA*	AR

	Familial Hemophagocytic Lymphohistiocytosis (FHL)	*PRF1*	AR
		UNC13D	AR
	Glucose-6-phosphate dehydrogenase deficiency	*G6PD*	XLD
Skeletal system diseases	X-linked dominant hereditary hypophosphatemic rickets	*PHEX*	XLD
Neuromuscular disease	Spinal muscular atrophy	*SMN1*	AR
	Pyridoxine-dependent epilepsy (PDE)	*ALDH7A1*	AR
	Hereditary spastic paraplegia (HSP)	*REEP1*	AD
		ATL1	AD
		SPAST	AD
		SPG11	AD
	Congenital myotonia	*CLCN1*	AD
	Duchenne muscular dystrophy (DMD)	*DMD*	XLR
	Tyrosine hydroxylase deficiency (THD)	*TH*	AR
	Glucose transporter 1 deficiency syndrome (GLUT1-DS)	*SLC2A1*	AD
	Ohtahara syndrome	*SCN1A*	AD
		PCHD19	AD
Endocrine disease	Congenital hypothyroidism (CH)	*DUOXA2*	AR
		DUOX2	AR
		TSHR	AR
	Congenital adrenal hyperplasia (CAH)	*CYP11B1*	AR
		CYP17A1	AR
	Kallmann syndrome (KS)	*ANOS1*	XLR
		FGFR1	AD
		PROKR2	AR/AD
		CHD7	AD
	X-linked adrenal hypoplasia congenit (X-AHC)	*NR0B1*	XLR
	Combined pituitary hormone deficiency type 2	*PROP1*	AR
	Permanent neonatal diabetes mellitus	*KCNJ11*	AD
		ABCC8	AD/AR
	Familial hyperinsulinemia	*ABCC8*	AD/AR
		KCNJ11	AR
		INSR	AD
Intrahepatic cholestasis	Progressive Familial Intrahepatic Cholestasis	*ATP8B1*	AR
		ABCB11	AR
		ABCB4	AR
Hearing disorder	Hereditary non-syndromic deafness	*SLC26A4*	AR
		GJB2	AR
		MYO15A	AR

		TMC1	AR
		TMPRSS3	AR
		OTOF	AR
		CDH23	AR
	Usher syndrome	*MYO7A*	AR
		PCDH15	AR
		USH2A	AR
	Mitochondrial non-syndromic sensorineural hearing loss (mNSSNHL)	*MT-RNR1*	
Immunodeficiency disease	Wiskott-Aldrich syndrome	*WAS*	XLR
	X-linked chronic granulomatous disease	*CYBB*	XLR
	X-linked angammaglobulinemia	*BTK*	XLR
	Severe combined immunodeficiency (SCID)	*IL2RG*	XLR
		RAG1	AR
	X-linked lymphoproliferative syndrom	*SH2D1A*	XLR
		XIAP	XLR
	Familial Mediterranean fever (FMF)	*MEFV*	AR
	X-linked hyperimmunoglobulin M syndrome (HIM)	*CD40LG*	XLD
	Severe congenial neutropenia (SCN)	*ELANE*	AD
Other metabolic related diseases	Bile Acid Synthesis Defect (BASD)	*HSD3B7*	AR
	Hepatolenticular degeneration (HLD)	*ATP7B*	AR
	Cerebrotendinous xanthomatosis (CTX)	*CYP27A1*	AR
	Menkes'disease (MD)	*ATP7A*	XLR
	Hypophosphatasia (HPP)	*ALPL*	AR/AD
Hereditary cancer related diseases	Retinoblastoma	*RB1*	AD
Other genetic diseases	Gitelman syndrome	*SLC12A3*	AR
	Leber hereditary optic neuropathy (LHON)	*MT-ND4*	
	X-linked Alport syndrome	*COL4A5*	XLD
	Alport syndrome	*COL4A3*	AD
		COL4A4	AR
	Tuberous sclerosis	*TSC1*	AD
		TSC2	AD
	Cystic fibrosis (CF)	*CFTR*	AR
	Co-Enzyme Q10 deficiency type 7	*COQ4*	AR

Abbreviations: AD, autosomal dominant inheritance; AR, autosomal recessive inheritance; XLD, x-linked dominant inheritance; XLR, x-linked recessive inheritance.

Table S4: Recommendations for follow-up and treatment initiation in presymptomatic individuals from this study.

No.	Disease	Follow-up (intervals)	Treatment initiation	Reference
1	Fabry disease	**Clinical evaluation:**<br>History, physical examination (including auxological evaluation) (annually).<br>**Laboratory tests:**<br>Blood count, urinalysis, 24-hour urine protein or 24-hour urine albumin/creatinine, serum creatinine, GFR estimated by formula, lipid profile (annually).<br>**Special tests:**<br>Pulmonary function testing, ophthalmologic examination, hearing test, pain and quality of life assessment (BPI, SF-36 Scale), ECG, echocardiogram (annually), 24-hour Holter monitor (only if ECG shows abnormalities, once every 1-2 years), cranial MRI and TCD (If no abnormalities, once every 2 years; if abnormalities, annually).	**Presymptomatic males with severe variants:**<br>If there's a family history, low α-Gal A activity, and high Lyso-GL-3 levels.<br>**Late-onset presymptomatic males/ females:**<br>If there is laboratory, histological, or imaging evidence of kidney, heart, or central nervous system involvement.	[44]
2	Krabbe disease (late onset)	**Clinical evaluation:**<br>History, physical examination (including auxological evaluation) (annually).<br>**Laboratory tests:**<br>Psychosine (if any signs or symptoms occur).<br>**Special tests:**<br>MRI brain, NCS, BAER, LP,VEP (if any signs or symptoms occur, otherwise every 2–5 years).	**Presymptomatic individuals:**<br>If any signs or symptoms appear along with abnormal laboratory, MRI, or NCS results.	[35]
3	GSD II	**Clinical evaluation:**<br>History, physical examination (including auxological evaluation) (annually).<br>**Laboratory tests:**<br>CK (annually)<br>**Special tests:**<br>**Musculoskeletal:** EMG, muscle CT, MRI, or ultrasound (every 1–2 years).<br>**Cardiovascular:** Chest X-ray, ECG, echocardiogram, cardiac MRI (every 1–2 years).<br>**Respiratory:** pulmonary function test and sleep monitoring (every 1–2 years).	**Presymptomatic individuals:**<br>If any signs or symptoms, including skeletal muscle weakness on examination and respiratory muscle involvement confirmed by pulmonary function tests.	[45,46]
4	NPD-A/B	**Clinical evaluation:**<br>History, physical examination (including auxological evaluation) (annually).<br>**Laboratory tests:**<br>Blood count, serum chemistries, lipid profile (annually).<br>**Special tests:**<br>**Respiratory:** Chest X-ray/CT (every 2–4 years).	**Presymptomatic individuals:**<br>If visceral or neurologic manifestations appear, treatment may be considered	[47,48]

		**Musculoskeletal:** assess for fractures and/or extremity pain (each visit).<br>**Neurological:** comprehensive neurologic evaluation, assess neurologic function and frequency of headaches (annually).<br>**Cardiovascular:** ECG, echocardiogram, coronary angiogram as indicated (adult only, every 3-5 years).<br>**Visceral:** ultrasonography or MRI to assess liver and spleen size (in response to physical examination).<br>**Developmental or cognitive:** document baseline degree of cognitive impairment including motor, adaptive, cognitive and speech/language (6 monthly in children, 12 monthly in adults).<br>**Neuropsychiatric:** document psychiatric manifestations (annually).		
5	MPS II	**Clinical evaluation:**<br>History, physical examination (including auxological evaluation) (annually).<br>**Special tests:**<br>**Neurological:** neurobehavioral assessment/cognitive testing, hand function tests (annually); MRI/CT of the head +/− gadolinium, LP measurement of CSF pressure, MRI cervical spine (every 1–3 years); Cervical spine flexion/extension (every 2–3 years, and before general anesthesia); Nerve conduction (at 4–5 years old, then at 1- or 2-year intervals).<br>**Cardiovascular:** ECHO/ECG, Holter (conduction irregularities) (annually)<br>**Musculoskeletal:** JROM (annually), X-ray (spine, hips and pelvis) (Upon diagnosis and thereafter in response to signs and symptoms).<br>**Respiratory:** pulmonary function (chest X-ray, oxygen saturation, sleep study to detect OSA, 6MWT, 3-minute stair climbing test) (Upon diagnosis or when patient is old enough to cooperate, then yearly); Sleep monitoring (every 3–5 years, then upon suspicion of OSA).<br>**Ophthalmologic:** Standard ophthalmologic examination (annually).<br>**Auditory:** otological and audiological examinations (every 6~12month)	**Presymptomatic individuals:**<br>If any signs or symptoms appear, seek treatment as early as possible.	[49]

ECG= electrocardiogram, CT= computed tomography, MRI= magnetic resonance imaging, TCD= transcranial doppler, NCS=nerve conduction study, Psy=psychosine, BAER=brainstem auditory evoked potential, LP=lumbar puncture, VEP = visual evoked potentials, CK=creatine kinase, EMG=electromyography, 6MWT= 6-minute walk test; CSF= cerebrospinal fluid, ECHO= echocardiogram; EEG= electroencephalography; JROM= joint range of motion; LP= lumbar puncture; OSA= obstructive sleep apnea.

Table S5: Carrier rate of LSDs.

No.	Disease	Gene	Carrier count	Carrier rate (1/n)
1	MPS VII	*GUSD*	10 (0.73%)	0.044% (1/2269)
2	MPS VI	*ARSB*	17 (1.24%)	0.075% (1/1335)
3	Sandhoff disease	*HEXB*	17 (1.24%)	0.075% (1/1335)
4	Tay-Sachs disease	*HEXA*	18 (1.32%)	0.079% (1/1260)
5	MPS IV-A	*GALNS*	30 (2.19%)	0.132% (1/756)
6	GM1 gangliosidosis	*GLB1*	37 (2.71%)	0.163% (1/613)
7	Metachromatic leukodystrophies	*ARSA*	55 (4.02%)	0.242% (1/412)
8	NPD-C	*NPC1/NPC2*	57 (4.17%)	0.251% (1/398)
9	MPS I	*IDUA*	60 (4.39%)	0.264% (1/378)
10	MPS III	*SGSH/NAGLU/HGSNAT*	110 (8.05%)	0.485% (1/206)
11	NPD-A/B	*SMPD1*	181 (13.24%)	0.798% (1/125)
12	GSD II	*GAA*	195 (14.26%)	0.860% (1/116)
13	Krabbe	*GALC*	580 (42.43%)	2.557% (1/39)
	All		1,367	1/17

Table S6: Cut-off value and reference range for lysosomal enzyme activity.

	**GALC**	**GAA**	**ASM**	**IDUA**	**GLA**
Median (μM/h)	2.15	6.09	1.96	2.22	7.78
Mean (μM/h)	2.25	6.75	2.14	2.34	8.19
0.2 MOM (μM/h)	0.43	1.22	0.39	0.44	2.33 (0.3MOM)
0.5percentile	0.7389	1.4467	0.99115	1.05005	3.6457
99.5percentile	4.82885	15.75905	6.4495	4.8699	16.60315

MOM, multiple of the median.

Table S7: Percentile position of enzyme activity in lysosomal storage disease carriers among negative samples.

	**GALC**		**GAA**		**ASM**		**IDUA**	
**Negative percentile**	**Enzyme activity (μmol/L/h)**	**Carrier percentage (%)**	**Enzyme activity (μmol/L/h)**	**Carrier percentage (%)**	**Enzyme activity (μmol/L/h)**	**Carrier percentage (%)**	**Enzyme activity (μmol/L/h)**	**Carrier percentage (%)**
10	1.289	48.18	3.675	33.99	1.3	51.28	1.45	42.22
20	1.55	62.27	4.64	50.98	1.518	59.83	1.688	48.89
30	1.737	72.73	5.157	60.13	1.637	64.96	1.87	55.56
40	1.94	81.36	5.692	67.32	1.816	72.65	2.086	68.89
50	2.15	84.55	6.09	70.59	1.96	76.07	2.215	73.33

Table S8: FRPs and PPVs in NBGS or Enzyme activity screening across different studies.

Disease	NBGS			Enzyme activity screening				
	**Screened newborns**	**FPR**	**PPV**	**Region**	**Screened newborns**	**FPR**	**PPV**	**Reference**
Krabbe	22,687	0%	100%	Shanghai of China	50,108	0.046%	28.13%	[10]
				New York of USA	550,000	0.004%	8%	[36]
GSD II	22,687	0.0132%	25%	Shanghai of China	50,108	0.584%	1.01%	[10]
				Taiwan of China	191,786	0.44%	1.83%	[11]
				Taiwan of China	402,281	1.03%	0.62%	[37]
				Taiwan of China	473,738	0.47%	1.25%	[38]
				Taiwan of China	132,538	0.82%	0.37%	[39]
				Japan	297,387	0.033%	7.34%	[40]
				Japan	103,204	0.215%	1.33%	[41]
				North East Italy	44,411	0.007%	25%	[8]

				Missouri of USA	43,702	0.023%	44.44%	[6]
				Hungary	40,024	0.14%	14.06%	[42]
				Austria	34,736	0.003%	80%	[43]
NPD-A/B	22,687	0%	100%	Shanghai of China	50,108	0.002%	83.33%	[10]
				Hungary	40,024	0.007%	40%	[42]
				Austria	34,736	0.003%	0%	[43]

Table S9: Potential onset periods of clinical symptoms for different LSDs.

No.	Disease	Approximate onset time of clinical symptoms
1	MPS I	Classic: 6 months to 1 year old<br>Mild: 3 to 10 years old
2	MPS II	After 18 months old
3	MPS III (A/B/C)	1 to 4 years old
4	MPS IV A	Severe: 1 to 3 years old<br>Mild: adolescence
5	MPS VI	Classic: 2 to 3 years old<br>Mild: after 10 years old
6	MPS VII	Severe: fetal period to 6 years old<br>Mild: childhood
7	GSD II	Infantile: before 1 year old<br>Late-onset: 1 to 60 years old
8	Metachromatic leukodystrophy	Infantile: before 2.5 years old<br>Adolescent: 4 to 16 years old<br>Adult: 15 to 62 years old
9	GM1 gangliosidosis	Early infantile: before 6 months old<br>Late infantile/ adolescent: 6 months to 8 years old<br>Adult: after 8 years old
10	Sandhoff disease	Infantile: about 6 months old<br>Adolescent: 1 to 18 years old<br>Adult: after 18 years old
11	Tay-Sachs disease	Infantile: 4 to 6 months old<br>Adolescent: 1 to 18 years old<br>Adult: after 18 years old

12	Krabbe disease	Early infantile: 3 to 6 months old
		Late infantile: 6 months to 3 years old
		Adolescent: 3 to 8 years old
		Adult: 10 to 35 years old
13	Fabry disease	Classic: childhood
		Late-onset: 40 to 70 years old
14	NPD-A/B	Type A: After 1 year
		Type B: After 2 years
15	NPD-C	Infantile: fetal period to 6 years old
		Adolescent: 6 to 15 years old
		Adult: after 15 years old

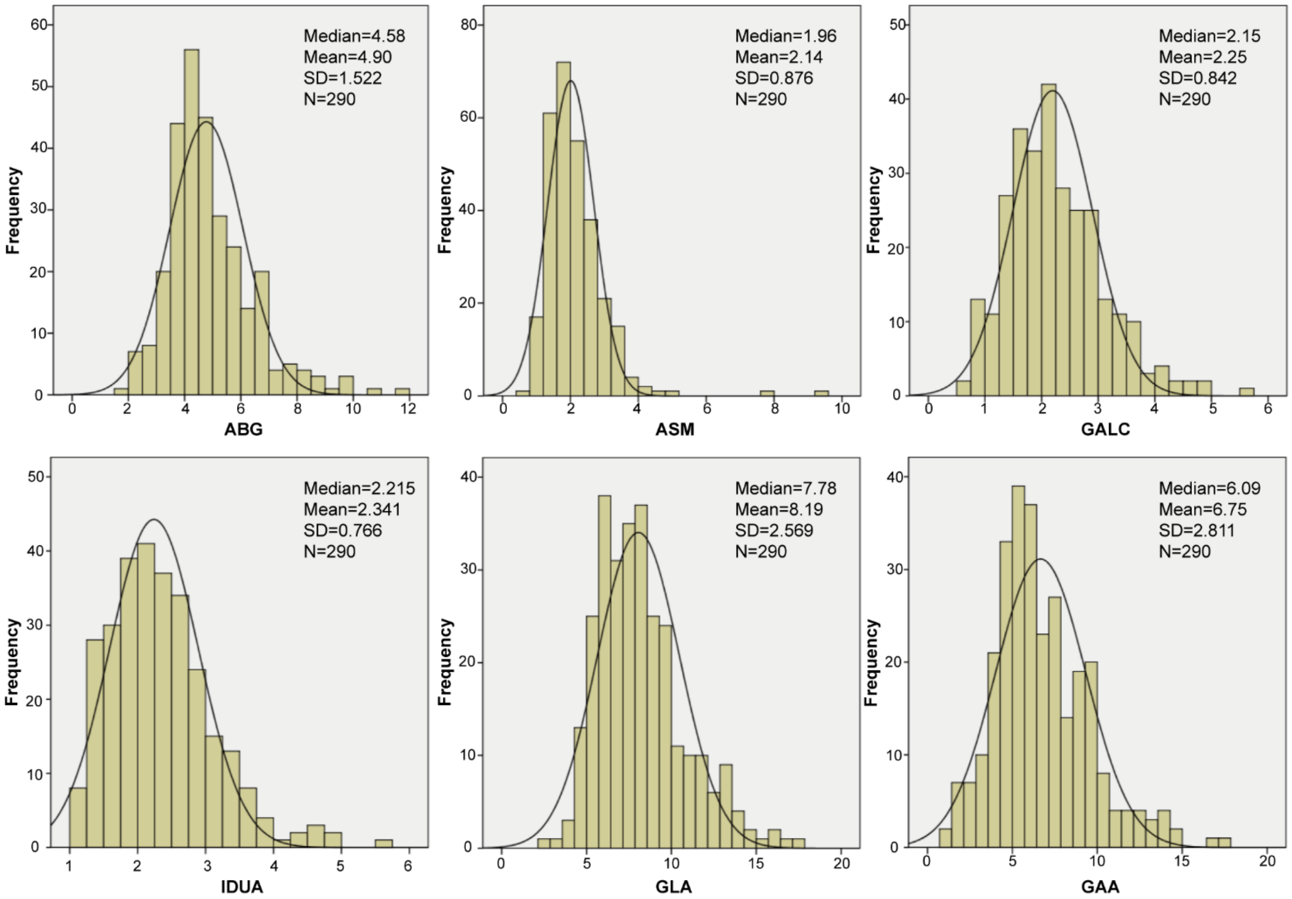

Fig S1. Histogram representation and statistical data of the distribution of enzymatic activity in healthy newborns.

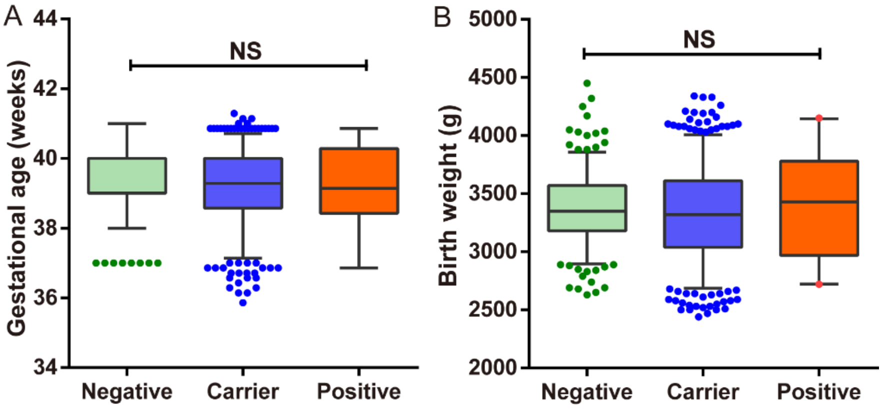

Fig S2. Statistical analysis of gestational age and birth weight in samples for enzyme activity detection. NS, not significant.

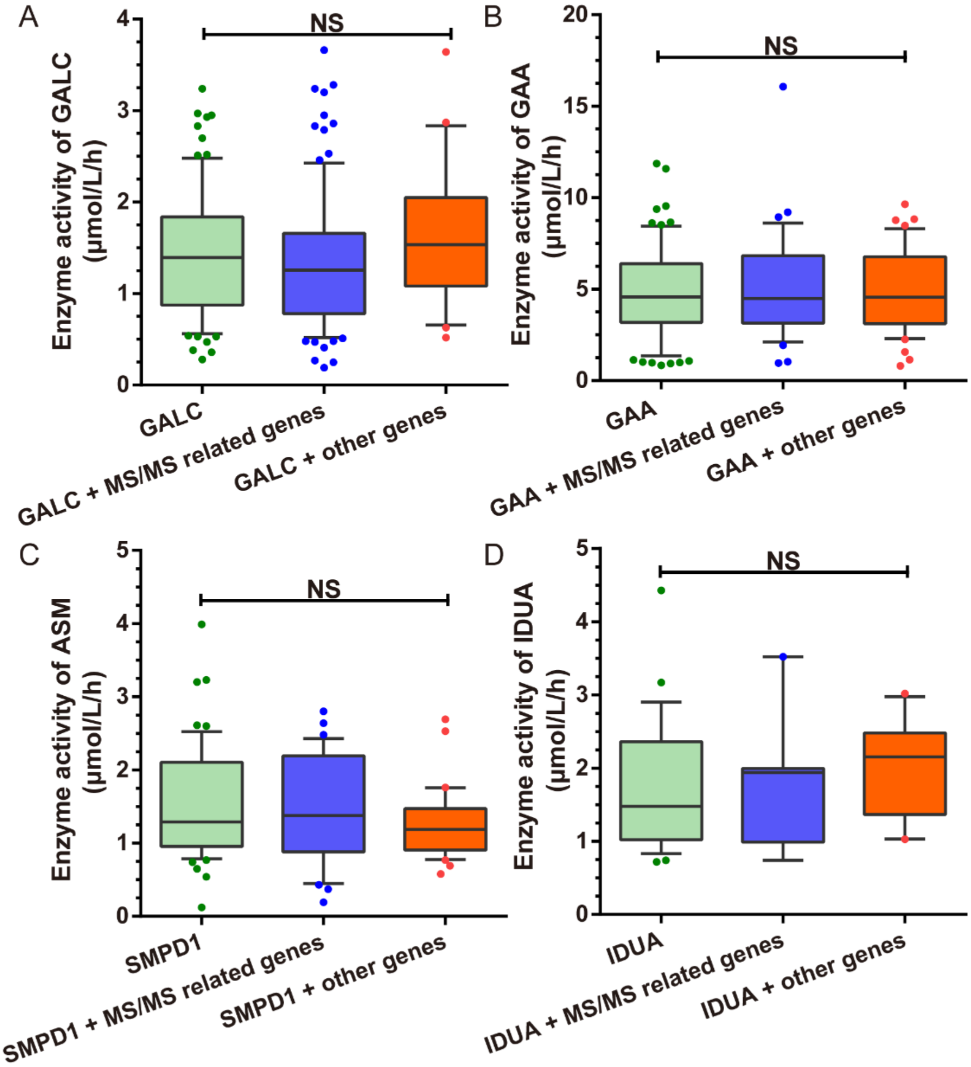

Fig S3. The influence of carrying both lysosomal storage disorders genes and other disease genes on enzyme activity results. (A) Carrying GALC and other genes. (B) Carrying GAA and other genes. (C) Carrying ASM and other genes. (D) Carrying IDUA and other genes. NS, not significant.
